# Provenance and Tectonic Controls in Eastern Junggar: Insights from Petrography and REE Geochemistry

**DOI:** 10.3390/molecules30163399

**Published:** 2025-08-18

**Authors:** Shengzhu Wang, Hongzhou Yu, Baosheng Li, Jinqi Han, Can Zhao, Yaoyun Guo, Jiaye Liu, Chang Su, Xu Chang, Tong Wu, Haoqing Huang

**Affiliations:** 1 Shandong Institute of Petroleum and Chemical Technology, Dongying 257061, China; wangshengzhu@sdipct.edu.cn; 2 Exploration and Development Research Institute, Shengli Oilfield, SINOPEC, Dongying 257015, China; 3 Ningcheng Meteorological Bureau, Inner Mongolia Autonomous Region, Chifeng 024200, China; 4 Inner Mongolia Engineering Research Center of Geological Technology and Geotechnical Engineering, Inner Mongolia University of Technology, Hohhot 010051, China; 5 Key Laboratory of Geological Hazards and Geotechnical Engineering Defense in Sandy and Drought Regions at Universities of Inner Mongolia Autonomous Region, Inner Mongolia University of Technology, Hohhot 010051, China; 6 Langfang Integrated Natural Resources Survey Center, China Geological Survey, Langfang 065000, China; 7 Research Center of Applied Geology of China Geological Survey, Chengdu 610036, China

**Keywords:** provenance discrimination, REE–trace element geochemistry, sandstone petrography, sedimentary tectonics, multivariate analysis

## Abstract

Rare earth elements (REEs) and trace elements, due to their relative stability during sedimentary processes, are effective geochemical proxies for sediment provenance. In the Dongdaohaizi Depression of the eastern Junggar Basin, the provenance of the Middle Jurassic Sangonghe Formation remains contentious. In this study, representative sandstone samples were systematically collected from all three members of the Sangonghe Formation in both the Dongdaohaizi Depression and its western margin. Through comprehensive petrographic and geochemical analyses, we obtained the following results. The Sangonghe Formation is primarily composed of feldspathic lithic sandstones, lithic sandstones, and minor lithic–feldspathic sandstones. The heavy mineral assemblage includes zircon, garnet, chromite, and rutile, suggesting source rocks of intermediate to acidic igneous, metamorphic, and mafic lithologies. The total REE contents range from 101.84 to 192.68 μg/g, with an average of 161.80 μg/g. The ∑LREE/∑HREE ratios vary from 6.59 to 13.25 (average 10.96), and the average δEu values are close to 1. The δCe value ranges from 1.09 to 1.13 (average 1.11). Trace element discrimination diagrams, including La-Th-Sc, Th-Co-Zr/10, Th-Sc-Zr/10, and La/Y-Sc/Cr ternary plots, indicate that most samples fall within the continental island arc domain, with a few plotting in the passive continental margin field. Comparison with potential surrounding source regions reveals dual provenances: an eastern source from the Kalamaili Mountains and a western source from the Zhayier Mountains. During the Early Jurassic, these two orogenic belts acted as distinct sediment sources. The Zhayier Mountains provided stronger input, with fluvial and tidal processes transporting sediments into the basin, establishing the primary subsidence center in the west of the depression. By the Middle Jurassic, continued thrusting of surrounding fold belts caused a migration of the lake center and the main depocenter to the western edge of the Dongdaohaizi Depression, while the former depocenter gradually diminished. Furthermore, sustained erosion and denudation of the Mosowan Uplift during the Early–Middle Jurassic reduced its function as a structural barrier, thereby promoting increased mixing between eastern and western sediment sources. The study not only refines existing paleogeographic models of the Junggar Basin, but also demonstrates the utility of REE–trace geochemistry in deciphering complex provenance systems in tectonically active basins.

## 1. Introduction

Understanding the provenance of clastic sediments is essential for reconstructing basin evolution, yet accurately distinguishing contributions from multiple source areas remains a key challenge in sedimentary geology [1,2,3]. Traditional techniques—such as paleocurrent measurements and compositional analysis—offer valuable insights but often fall short when source rocks share similar lithological characteristics or when sediment recycling obscures primary signals [1,4,5]. In such complex geological settings, rare earth elements (REEs) and trace elements provide a robust geochemical toolkit, owing to their relative immobility during sedimentary processes and their sensitivity to source rock composition and tectonic regime [6,7,8]. Their systematic fractionation patterns and diagnostic elemental ratios offer unique insights into sediment origins, enabling discrimination between various source rock types and tectonic settings [9].

The effectiveness of REE-based provenance studies lies in their ability to reflect both source rock composition and tectonic environment [6,10,11]. For instance, felsic rocks typically exhibit distinct negative Eu anomalies and enriched light REE patterns, while mafic rocks show relatively flat heavy REE distributions and less pronounced Eu anomalies [10,12,13]. Furthermore, specific trace element ratios can reveal sediment recycling processes and mixing histories [8]. These geochemical indicators have proven valuable in diverse geological settings, from active continental margins to stable cratonic regions.

The Junggar Basin in northwestern China, formed during the closure of the Paleo-Asian Ocean, records a complex history of oceanic subduction, terrane amalgamation, and intracontinental deformation [14,15,16,17,18,19]. Along its eastern margin lies the Dongdaohaizi Depression, a key sub-basin preserving Mesozoic sedimentary sequences that chronicle this tectonic evolution [20,21,22]. Among these, the Middle Jurassic Sangonghe Formation remains underexplored in terms of high-resolution provenance studies.

Previous work in the Junggar Basin has relied heavily on sandstone petrography and detrital zircon U–Pb geochronology [23,24,25]. Although informative, these approaches have led to conflicting interpretations regarding the Sangonghe Formation, particularly concerning the relative influence of northern orogenic belts versus more distal cratonic sources [26,27].

Early petrographic studies suggested that the Sangonghe Formation is dominated by lithic sandstones, with volcanic lithic fragments being the most abundant, indicating a proximal volcanic arc provenance [28,29,30]. However, this interpretation conflicts with subsequent geochemical analyses [31,32]. Whole-rock La/Sc ratios point toward a felsic source region, yet the sandstone samples contain more than 20% mafic lithic fragments—an apparent inconsistency. Later studies clarified that these mafic fragments are, in fact, altered basalts rather than components of the original source rocks. This finding underscores the necessity of integrating petrographic observations with rare earth element (REE) geochemistry and single-mineral analyses to obtain a more accurate understanding of provenance. In a separate study focusing on detrital zircon geochronology, the Sangonghe Formation exhibits prominent age peaks at ~300 Ma (corresponding to the Tianshan Orogenic Belt) and ~450 Ma (Altai basement), leading to the interpretation that the primary source was the North Tianshan region [33,34]. However, the ~450 Ma age peak could alternatively reflect contributions from the Paleozoic Altai basement or recycled sediments from within the Junggar Block itself, such as eroded Carboniferous strata [35,36]. Notably, this study did not conduct further geochemical analyses, such as Th/U ratio screening of zircon grains, and lacks trace element data to support its provenance interpretations. Moreover, the role of sediment recycling and tectonically induced sediment routing changes remains poorly constrained.

To address these questions, this study combines petrographic examination with REE and trace element geochemistry of the Sangonghe Formation sandstones. Unlike single-mineral techniques, bulk sediment geochemistry provides integrated information about the entire source terrain, making it particularly suitable for studying complex sedimentary systems. The results not only refine the provenance model for the Dongdaohaizi Depression but also contribute to our understanding of the Mesozoic tectonic–sedimentary evolution in the Junggar Basin.

## 2. Geological Setting

**Figure 1:**
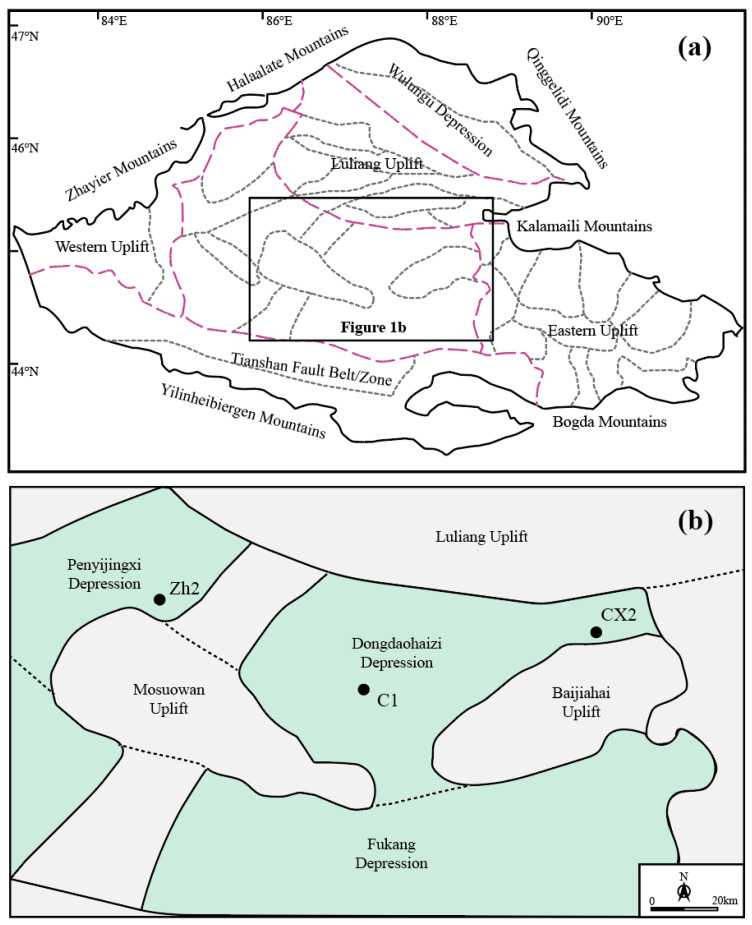
(**a**) Geological map of Junggar Basin. (**b**) Geological map of Dongdaohaizi area. Black points indicate the sampling location in this study.

The basin occupies a pivotal tectonic position in northwestern China, representing a key junction where the Kazakhstan, Siberian, and Tarim plates converge (Figure 1a) [17,37,38]. Its triangular geometry and pronounced structural zonation reflect a complex accretionary history. Among its major tectonic units, the Dongdaohaizi Depression represents a second-order structural subunit within the Central Depression, bounded by the Mosuowan and Baijiahai uplifts to the west and east, respectively (Figure 1b). These uplift zones are considered important potential source regions for Mesozoic clastic sediments.

The geological evolution of the Dongdaohaizi Depression began during the Early Hercynian orogeny, with initial marine deposition of shales and marls accompanied by calc-alkaline volcanism [17,39,40]. From the Early Carboniferous to Early Permian, the region experienced intensive magmatic–tectonic activity, leading to the emplacement of granitic intrusions and extensive volcanism—particularly in the surrounding orogenic belts (e.g., the Zhaheba and Karamaili belts). These lithologies are now considered potential felsic and intermediate sources for Jurassic detritus. The Late Hercynian and Yanshanian deformation episodes reactivated existing faults and promoted uplift and erosion, shaping the modern structural grain and influencing sediment routing [41,42,43].

By the Early Jurassic, the Junggar Basin had evolved into a predominantly continental interior setting, with sediment input largely governed by peripheral orogenic belts. During deposition of the Sangonghe Formation, NE–SW trending normal faults facilitated accommodation space, while the adjacent Karamaili Belt (to the northeast) and Baijiahai Uplift (to the east) likely served as primary sediment sources. The Karamaili Belt is particularly significant, consisting of deformed ophiolitic mélanges, volcanic arc assemblages, and granitoids—features that would impart distinctive geochemical fingerprints to the derived sediments.

**Figure 2:**
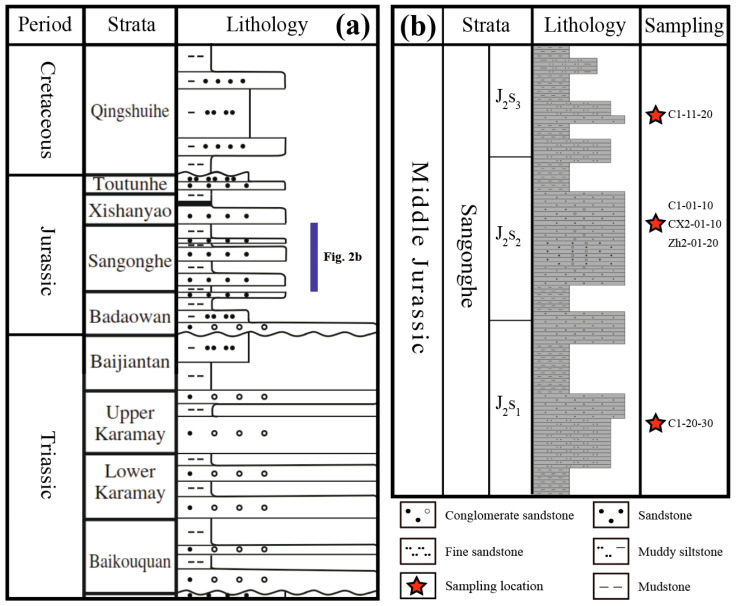
(**a**) Mesozoic chronostratigraphy and lithology section of Junggar Basin. (**b**) Chronostratigraphy and lithology section of Middle Jurassic Sangonghe Formation in Dongdaohaizi Depression. Abbreviations J_2_s_1_, J_2_s_2_ and J_2_s_3_ indicate the lower, middle and upper members of the Middle Jurassic Sangonghe Formation.

Stratigraphically, the Jurassic succession in the Dongdaohaizi Depression includes the Badaowan, Sangonghe, Xishanyao, and Toutunhe formations (Figure 2a). The Sangonghe Formation—the focus of this study—is interpreted to have formed in a fluvio-deltaic system with varying proximity to basin margins. Its three members show distinct lithofacies: the lower member (~40 m) comprises thick-bedded grey pebbly sandstones interbedded with mudstones, interpreted as proximal fluvial or braided river deposits; the middle member (~170 m) contains finer-grained sediments, including silty mudstones and coal seams, indicating a more distal, low-energy swamp to floodplain environment; the upper member (~30 m) returns to coarser fluvial deposition with pebbly sandstones and conglomerates, especially near basin margins. These lithofacial transitions reflect shifting paleogeographic and tectonic controls, possibly linked to fluctuating uplift and erosion rates in adjacent source terranes.

To capture this variability, representative sandstone samples were collected from multiple stratigraphic levels and geographic positions across the depression. Wells C1 (all three members), CX1 (middle member), and Zh2 (middle member) were selected to ensure both vertical and lateral representation (Figure 1b and Figure 2b). These samples form the basis for integrated petrographic and geochemical analyses aimed at delineating sediment provenance and reconstructing Early Jurassic sediment dispersal systems.

## 3. Results

### 3.1. Composition of Rock Fragments

The petrographic analysis of clastic components in the study area shows a good correspondence with the lithological characteristics of the potential source rocks. Based on thin section analyses of the Sangonghe Formation in both the main study area and the western part of the Dongdaohaizi Depression, the lithological composition has been systematically summarized (Table A1).

**Figure 3:**
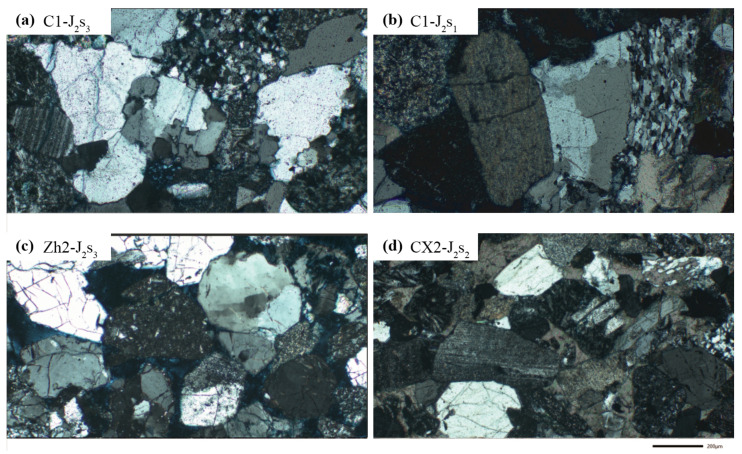
Cross-polarized light photomicrograph of sandstone samples from Sangonghe Formation.

In terms of lithological composition, the Sangonghe Formation in the study area is mainly composed of feldspathic lithic sandstones, lithic sandstones, and minor lithic–feldspathic sandstones. These rocks are characterized by a high content of volcanic and metamorphic lithic fragments. In the western region, the lithology is predominantly feldspathic lithic sandstone and lithic-rich quartz sandstone, with subordinate lithic–feldspathic sandstone and lithic sandstone (Figure 3).

Samples from Well C1, primarily from the second member of the Sangonghe Formation within the Dongdaohaizi Depression, show abundant volcanic and metamorphic lithic fragments and exhibit low compositional maturity. This suggests a short transport distance, indicating a proximal source area controlled by nearby orogenic activity. Well CX2, with fewer samples, also contains lithic sandstones dominated by volcanic lithics, and similarly shows low maturity, again implying limited sediment transport. Overall, samples from the western Dongdaohaizi region reflect a dominance of volcanic lithic fragments with low maturity, consistent with short transport distances. Although both Wells CX2 and C1 show similar maturity, the slightly higher maturity in CX2 implies a possible eastern source. In contrast, Well Zh2 appears to have a dominant western source influence.

Under the microscope, quartz content in Well C1 is relatively high, with abundant tuffaceous lithics and minor basaltic fragments observed (Figure 3). Vertically, both total lithic and tuffaceous lithic contents in the Sangonghe Formation of Well C1 decrease upward, while porosity increases.

According to microscopic petrographic features and Dickinson’s provenance discrimination model, the overall source area for the Sangonghe Formation in the study area is characterized by a recycled orogen provenance, particularly a transitional recycled zone derived from collision–suture and fold–thrust belts [44,45]. The tectonic background in the western region is largely similar to that of the main study area.

### 3.2. Heavy Mineral Analysis Results

**Figure 4:**
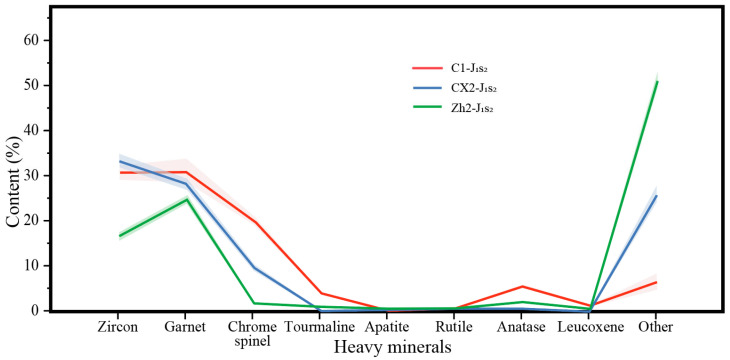
Relative contents of heavy minerals of samples from the Sangonghe Formation. Solid lines represent averaged data, and shadows represent ranges of all data. All data are shown in Table A2.

There are marked differences in heavy mineral assemblages between the Sangonghe Formation in the study area and its western counterpart. The mineral compositions within the depression clearly differ from those outside it to the west (Table A2, Figure 4).

The heavy mineral suite in Well C1 consists mainly of zircon, garnet, chromite, and rutile, indicative of source rocks dominated by intermediate to acidic igneous, metamorphic, and basic igneous lithologies [46,47]. Well CX2 exhibits a similar assemblage—zircon, garnet, and chromite—also pointing to similar source lithologies. In contrast, the assemblage in Well Zh2 is largely dominated by garnet and zircon, suggesting a provenance mainly composed of metamorphic and intermediate-acid igneous rocks [48,49,50]. The similarity between C1 and CX2—especially their high chromite content—further supports a shared provenance. In comparison, chromite is significantly less abundant in Well Zh2, highlighting a distinct difference in sediment sources between the western and inner depression areas.

From the Badaowan to the Sangonghe Formations, notable changes in heavy mineral assemblages occur in both the study and western regions. An increased abundance of garnet in the study area suggests ongoing uplift and denudation of the Kelameili Mountains, with deeper metamorphic units becoming progressively exposed. Conversely, the Sangonghe Formation in Well Zh2 appears to have been influenced by an eastern source.

Compared to previously published data on the Badaowan Formation, heavy mineral contents such as zircon, garnet, chromite, and tourmaline in Well Zh2 have significantly decreased, while authigenic minerals such as barite and pyrite have increased, indicating lower compositional maturity and altered heavy mineral assemblages. This shift suggests that eastern sources may have contributed to the Zh2 sedimentary record. Meanwhile, the high zircon content in the study area implies higher compositional maturity and longer transport distances, whereas the increased garnet content reflects continuous uplift and exposure of deeper metamorphic basement rocks, pointing to an expanding provenance area.

Quantitative indices such as the garnet–zircon index (GZi = 48–73) and apatite–tourmaline index (ATi = 32–58) were calculated, with statistical uncertainty <5%. These values confirm differences in compositional maturity and source lithology. GZi increases upward in Well C1, consistent with progressive unroofing of deeper metamorphic units in the Kelameili region.

### 3.3. Geochemical Analysis

#### 3.3.1. Rare Earth Element (REE) Geochemistry

##### REE Abundance and Characteristic Ratios

The total REE contents of the Sangonghe Formation in Well CX2 range from 101.84 to 192.68 μg/g, with an average of 161.80 μg/g, with analytical uncertainty <3% RSD. In Well C1, values range from 102.96 to 191.30 μg/g, averaging 130.45 μg/g. Western samples from the Dongdaohaizi Depression yield a broader range of 77.43 to 247.45 μg/g, with an average of 160.63 μg/g. Overall, REE concentrations in Well C1 are slightly lower than the average upper continental crust value (146.4 μg/g), whereas CX2 and western samples are generally higher (Table 1).

The ∑LREE/∑HREE ratio in CX2 ranges from 6.59 to 13.25 (avg. 10.96), in C1 from 6.40 to 7.95 (avg. 7.21), and in the western samples from 6.46 to 9.84 (avg. 8.26). Notably, the ratio in C1 is slightly below that of North American Shale Composite (7.44), suggesting relative LREE depletion and HREE enrichment. CX2 and western samples have higher ratios, indicating less pronounced LREE–HREE fractionation.

LaN/YbN values in CX2 are higher, implying steeper REE patterns, while values in C1 and western samples are closer to unity, indicating flatter patterns. LaN/SmN ratios are relatively consistent across regions, showing minimal internal LREE fractionation. Similarly, GdN/YbN ratios hover around 1, suggesting negligible HREE fractionation across the study and western regions.

The average δEu values for Sangonghe samples are close to 1, though most samples show slight negative Eu anomalies. δCe values range from 1.09 to 1.13 in CX2 (avg. 1.11), 0.96 to 1.03 in C1 (avg. 0.97), and around 1 in western samples, indicating a predominantly reducing depositional environment. Ce_anom values above −0.1 across all samples further support an overall anoxic to suboxic water column during sedimentation [51,52,53].

##### REE Normalized Distribution Patterns

**Figure 5:**
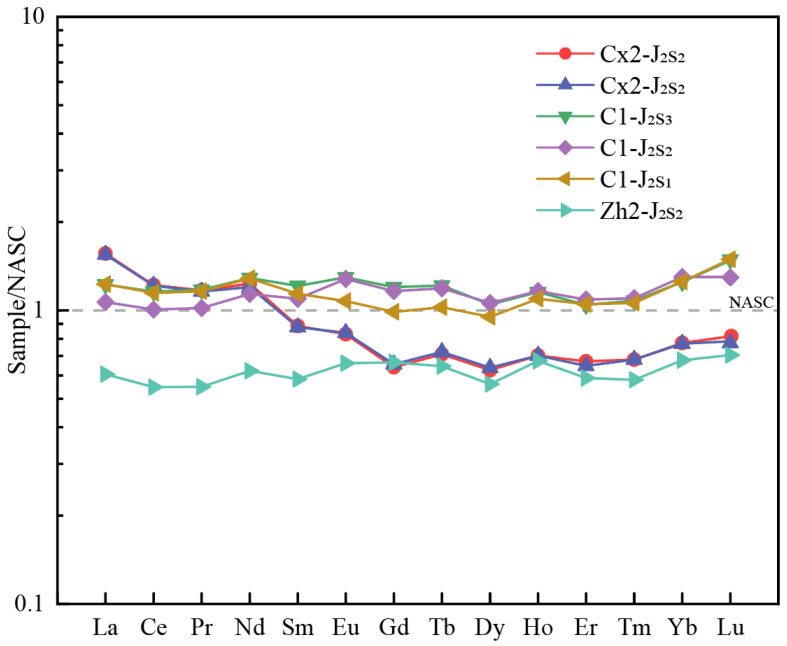
NASC-normalized REE patterns of samples from Sangonghe Formation. Solid icons represent averaged data, and shadows represent all data. All data are shown in Table A3.

The REE distribution patterns of C1 and CX2 samples show a generally flat trend with low LREE and HREE fractionation (Figure 5). A few CX2 samples display slight LREE enrichment and HREE depletion, forming right-tilted profiles.

Western samples from the Dongdaohaizi Depression also exhibit flat REE distribution curves with low internal fractionation, indicative of uniform REE behavior.

Despite some general similarities, the differences in REE distribution patterns between the study area and the west are significant, pointing to distinct provenance signatures.

According to Bhatia et al. (1983, 1986) [54,55], who analyzed REE signatures of greywackes from various tectonic settings, the REE characteristics of the Sangonghe Formation in both CX2 and C1 wells are most consistent with a continental island arc provenance. Similarly, REE patterns in Well Zh2 also align closely with a continental island arc setting. Thus, both the study and western areas appear to share the same broad tectonic framework.

#### 3.3.2. Trace Element Geochemistry

**Figure 6:**
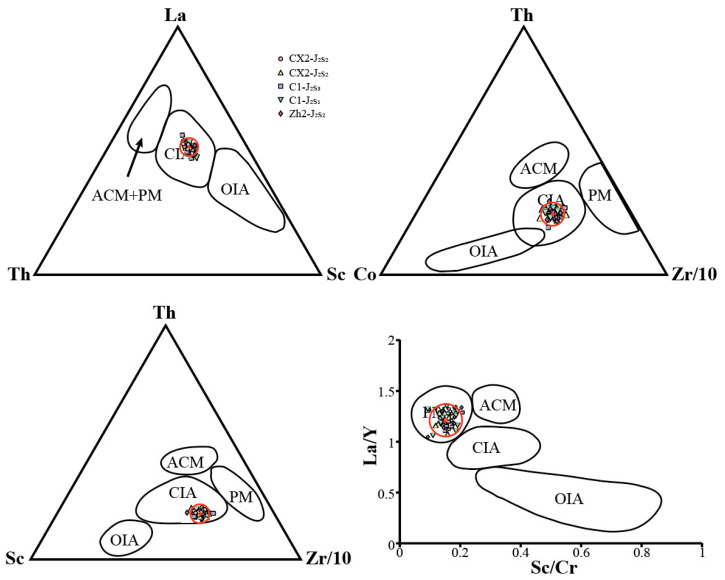
Identification diagrams of tectonic settings of samples from the Sangonghe Formation in the Dongdaohaizi Depression. ACM = active continental margin, PM = passive continental margin, CIA = continental island arc, OIA = oceanic island arc. All data are shown in Table 2. Red circles represent 95% confidence interval.

Trace element data from the study area have been plotted onto various tectonic discrimination diagrams (Figure 6) [55,56,57]. On the La-Th-Sc ternary plot, data points from both C1 and CX2 wells fall predominantly within the continental island arc field. The Th-Co-Zr/10 and Th-Sc-Zr/10 diagrams also place the samples within this tectonic regime. Similarly, the La/Y vs. Sc/Cr plot shows most points within the continental island arc domain, with a few falling into the passive continental margin field. Confidence ellipses (95%) were added to each group to assess grouping significance.

**Table 2:** Geochemical analysis data of trace elements in samples from the Sangonghe Formation.

Strata	Sample	La	Th	Sc	Co	Zr/10	Sc/Cr	La/Y
CX2-J_2_s_2_	C1-01	0.49	0.19	0.26	0.28	0.57	0.16	1.16
	C1-02	0.48	0.19	0.29	0.32	0.55	0.19	1.18
	C1-03	0.48	0.19	0.27	0.27	0.54	0.18	1.19
	C1-04	0.50	0.20	0.23	0.27	0.47	0.17	1.26
	C1-05	0.47	0.20	0.30	0.33	0.51	0.12	1.24
	C1-06	0.52	0.20	0.30	0.28	0.51	0.16	1.32
	C1-07	0.47	0.19	0.26	0.28	0.52	0.14	1.21
	C1-08	0.48	0.20	0.25	0.27	0.52	0.16	1.12
	C1-09	0.50	0.20	0.24	0.34	0.53	0.15	1.15
	C1-10	0.46	0.19	0.29	0.29	0.51	0.18	1.28
CX2-J_2_s_2_	C1-11	0.49	0.20	0.27	0.28	0.55	0.13	1.29
	C1-12	0.49	0.19	0.25	0.30	0.53	0.17	1.24
	C1-13	0.49	0.19	0.29	0.30	0.54	0.12	1.18
	C1-14	0.47	0.18	0.28	0.40	0.54	0.14	1.22
	C1-15	0.49	0.20	0.26	0.32	0.59	0.11	1.16
	C1-16	0.48	0.19	0.27	0.26	0.59	0.15	1.18
	C1-17	0.50	0.20	0.31	0.30	0.59	0.12	1.33
	C1-18	0.48	0.19	0.27	0.28	0.51	0.16	1.19
	C1-19	0.50	0.20	0.26	0.31	0.57	0.15	1.29
	C1-20	0.49	0.21	0.29	0.33	0.47	0.16	1.08
C1-J_2_s_3_	C3-01	0.49	0.18	0.27	0.32	0.51	0.17	1.21
	C3-02	0.48	0.21	0.28	0.26	0.55	0.16	1.28
	C3-03	0.49	0.20	0.25	0.29	0.53	0.21	1.29
	C3-04	0.48	0.18	0.29	0.24	0.54	0.14	1.23
	C3-05	0.49	0.20	0.30	0.33	0.53	0.14	1.23
	C3-06	0.49	0.20	0.25	0.29	0.54	0.15	1.18
	C3-07	0.48	0.17	0.29	0.32	0.49	0.15	1.16
	C3-08	0.51	0.18	0.21	0.36	0.54	0.15	1.11
	C3-09	0.47	0.21	0.32	0.32	0.55	0.17	1.15
	C3-10	0.51	0.19	0.29	0.36	0.57	0.16	1.20
C1-J_2_s_1_	C3-01	0.49	0.19	0.24	0.30	0.52	0.13	1.18
	C3-02	0.50	0.20	0.29	0.30	0.52	0.18	1.29
	C3-03	0.47	0.20	0.33	0.35	0.57	0.14	1.24
	C3-04	0.50	0.18	0.26	0.33	0.54	0.18	1.26
	C3-05	0.48	0.20	0.30	0.26	0.54	0.19	1.16
	C3-06	0.49	0.17	0.29	0.30	0.55	0.18	1.11
	C3-07	0.49	0.19	0.28	0.28	0.53	0.11	1.06
	C3-08	0.48	0.18	0.31	0.30	0.54	0.15	1.35
	C3-09	0.50	0.18	0.30	0.35	0.52	0.19	1.30
	C3-10	0.48	0.20	0.27	0.32	0.48	0.09	1.32
Zh2-J_2_s	C3-01	0.49	0.20	0.33	0.29	0.48	0.15	1.21
	C3-02	0.50	0.21	0.25	0.25	0.50	0.18	1.18
	C3-03	0.47	0.20	0.30	0.32	0.53	0.20	1.34
	C3-04	0.50	0.19	0.23	0.30	0.55	0.18	1.24
	C3-05	0.49	0.20	0.27	0.37	0.57	0.14	1.30
	C3-06	0.48	0.19	0.27	0.31	0.57	0.18	1.34
	C3-07	0.49	0.19	0.30	0.32	0.55	0.14	1.14
	C3-08	0.50	0.17	0.29	0.31	0.57	0.18	1.34
	C3-09	0.48	0.20	0.30	0.32	0.55	0.09	1.05
	C3-10	0.50	0.19	0.27	0.27	0.54	0.15	1.19

All four tectonic discrimination diagrams yield consistent results, supporting the conclusion that the Sangonghe Formation in the study area developed within a tectonic setting characterized by a combination of passive margin and continental island arc environments.

#### 3.3.3. Multivariate Statistical Analysis Results

The PCA revealed systematic covariation among petrological–mineralogical, heavy-mineral abundances and geochemical parameters. PC1 (49.6% variance) was strongly loaded by zircon (Zr: 0.92), garnet (Grt: 0.88), rutile (Rt: 0.85), and the garnet–zircon index (GZi: 0.88), reflecting a dominant control by resistate mineral fertility (Table A4). Samples with high PC1 scores (e.g., C1: 1.71) exhibited enrichment in these minerals, consistent with felsic source inputs, whereas low-PC1 samples (e.g., Zh2: −1.55) indicated mafic or recycled sediment dominance (Table A5).

PC2 (35.2% variance) correlated with REE systematics, including (La/Yb)N (0.85) and ∑REE (0.78), as well as the apatite–titanite index (ATi: 0.82), suggesting a secondary control by REE fractionation and accessory-phase dissolution during transport. For instance, CX2 (PC2 = 1.46) showed elevated LREE/HREE ratios linked to apatite preservation.

PC3 (8.96% variance) was driven by δEu (0.90) and tourmaline (Tur: 0.72), potentially signaling local hydrothermal alteration or plagioclase breakdown. Cross-validation with trace-element ratios (e.g., Zr/Sc vs. Th/Sc; R^2^ = 0.76) confirmed that PCA groupings align with independent provenance proxies.

The PCA statistically links heavy-mineral assemblages (e.g., Zr-Grt-Rt) to bulk geochemistry (e.g., ∑REE, Th/Sc), supporting our stratigraphic model of provenance shifts from felsic (high PC1) to mixed-mafic sources (low PC1/PC2).

## 4. Discussion

### 4.1. Provenance Analysis from Comparison

The Middle Jurassic in the Junggar Basin represents a crucial phase of basin formation and orogenic activity [14,58,59]. Situated at the southern margin of the Paleo-Asian tectonic domain, the Junggar Basin is an integral part of the Central Asian Orogenic Belt (CAOB) [14,60,61]. It is bounded on three sides by Paleozoic suture zones and fold belts that serve as major sediment sources. The western margin is delineated by the NE to ENE-trending West Junggar fold-and-thrust belt, including the Halaalate and Zhayier Mountains. The northeastern and eastern boundaries are defined by the Altay and East Junggar orogenic belts, particularly the Qinggelidi and Kalamaili Mountains. The southern margin is bordered by the North Tianshan fold belt and the Bogda Mountains [62,63,64].

Our results suggest that the Dongdaohaizi Depression received sediments transported over relatively short distances, indicating a dominant influence from nearby orogenic belts. While the CX2 and C1 wells exhibit similar mineral maturity, CX2 shows slightly higher maturity, implying a more distal or eastern sediment source. Given the basin’s topography and surrounding structural framework, we infer that sediments of the Sangonghe Formation in the Dongdaohaizi Depression were primarily sourced from the Kalamaili Mountains to the east. Conversely, the provenance of the Zh2 well appears to be dominantly influenced by the Zhayier Mountains in the west.

Heavy mineral assemblages display marked contrasts, further supporting the notion of distinct sediment source systems between the Dongdaohaizi Depression and its western periphery. Comparative analysis with surrounding orogenic belts reveals that uplifted sections of the Kalamaili Mountains expose significant ophiolitic sequences, including metamorphosed peridotites, gabbros, diabases, and mafic volcanics. These lithologies weather to yield zircon, chromite, tourmaline, and garnet—mineral components that are notably consistent with those identified in the Dongdaohaizi Sangonghe Formation [65,66,67]. In contrast, the heavy mineral assemblages west of the depression are more closely aligned with those reported from the Zhayier Mountains. Hence, both heavy mineral evidence and spatial variation support the dual-provenance model, with the eastern Kalamaili Mountains supplying sediments to the depression and the western Zhayier Mountains influencing the Zh2 region [68,69,70].

Rare earth element (REE) data indicate minor fractionation between light and heavy REEs but reveal low pattern similarity between eastern and western sediment sources, highlighting compositional differences. Trace element data show signatures consistent with both passive continental margin and continental island arc settings. Based on regional geological context, REE characteristics in the Kalamaili Mountains exhibit pronounced LREE/HREE fractionation, weak Eu anomalies, and enrichment in high field strength elements (HFSEs) [66,71,72,73,74,75]. These features closely match the geochemical signatures of sandstones from the Dongdaohaizi Sangonghe Formation. In contrast, West Junggar samples exhibit higher total REE contents (195.16 to 249.18 ppm), elevated LREEs (125.76 to 170.99 ppm), and HREEs (69.4 to 80.83 ppm), with steep LREE slopes and flatter HREE segments, and mild negative Eu anomalies, forming a right-leaning “V” shape pattern in chondrite-normalized REE plots [76,77,78,79,80]. These patterns, though broadly similar in total REE content, differ in Eu anomalies from Dongdaohaizi sediments but match the western Zh2 samples. Therefore, REE and trace element data reaffirm the eastern provenance from the Kalamaili Mountains and western provenance from the Zhayier Mountains.

Integrating previous findings, we note that heavy mineral assemblages in the Dongdaohaizi Depression underwent notable changes from the Early to Middle Jurassic, reflecting continuous uplift and exhumation of metamorphic rocks from the Kalamaili source area [22,81,82,83]. In the Zh2 well, the decrease in zircon, garnet, chromite, and tourmaline, along with increased authigenic barite and pyrite and lower maturity, suggests a reduced influence from the Kalamaili source and possible input from alternative western sources. Meanwhile, the overall high zircon content and increased garnet abundance in the Dongdaohaizi region imply a sustained uplift and unroofing of deeper metamorphic sequences in the Kalamaili Mountains. Compared to the Early Jurassic, sediment maturity increased in the Middle Jurassic, reflecting extended transport distances and an expanded source region [84,85,86].

Middle Jurassic sediments in the Sangonghe Formation indicate derivation from recycled orogenic sources with transitional recycled orogen signatures, typical of collision and fold–thrust belt environments [87,88,89,90]. Despite similar tectonic backgrounds in the western basin, the influence of two discrete source areas led to a mixture of distinct provenance signatures.

Additionally, the chemical index of alteration values in this area indicates minimal chemical weathering or post-depositional alteration, reducing the likelihood that geochemical trends reflect diagenetic modification. The lack of significant Neoproterozoic to Cambrian zircon age populations also argues against a dominant recycled cratonic component. Detrital zircon U–Pb age spectra from adjacent wells show a major age peak between 420 and 360 Ma, consistent with the magmatic history of the Kalamaili arc, further substantiating this provenance link.

Thus, we conclude that during the Middle Jurassic Sangonghe stage, the Dongdaohaizi Depression was primarily fed by detritus from the eastern Kalamaili Mountains, while its western segment received sediments from the Zhayier Mountains, reflecting a two-source sediment mixing system. From the Early to Middle Jurassic, the eastern Kalamaili source intensified and expanded westward, increasingly influencing the entire depression.

### 4.2. Provenance Interpretation from Statistics

**Figure 7:**
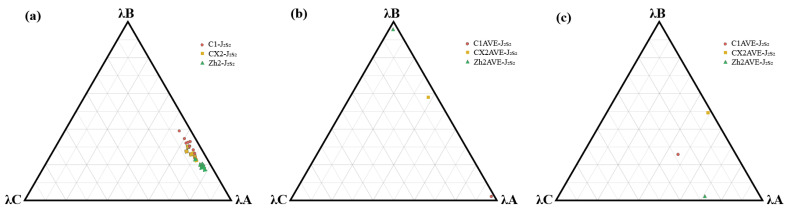
Ternary diagram of (**a**) petrological–mineralogical, (**b**) heavy mineral, and (**c**) REE data with weight contributions from three major provenances (λA: Kalamaili Mountains, λB: Zhayier Mountains, λC: Tianshan Mountains). All data are shown in Table A6, Table A7, Table A8, Table A9, Table A10 and Table A11.

The quantitative three-end-member mixing analysis provides critical insights into the sediment sources of the Sangonghe Formation within and around the Dongdaohaizi Depression. By projecting the normalized detrital compositions of 30 sandstone samples, heavy mineral compositions and REE of 3 averaged datasets, we derived the proportional contributions from three geological source regions: Kalamaili Mountains, Zhayier Mountains, and Tianshan Mountains (Figure 7; Table A6, Table A7, Table A8, Table A9, Table A10 and Table A11). This also provides assistance for the provenance interpretation and the restoration of ancient geography.

The detrital mixing model reveals systematic spatial variations in provenance between wells located within the depression (C1 and CX2) and the external well (Zh2) (Figure 7a; Table A6 and Table A7). For the C1 well samples (samples 1–10), the calculated average contribution was λ_A = 0.67, λ_B = 0.26, and λ_C = 0.07, indicating a dominant input from the Kalamaili Mountains, supplemented by a moderate Zhayier component and minimal input from Tianshan. Similarly, CX2 well samples (samples 11–20) show a comparable source pattern, with slightly more influence from Zhayier: λ_A = 0.61, λ_B = 0.31, λ_C = 0.08. The small but consistent Tianshan component across both internal wells suggests only limited long-distance sediment transport or minor tectonic influence from the south during this period. In contrast, Zh2 well samples, located to the west of the depression, exhibit a markedly different provenance signature. The mixing model yields a rather lower λ_A = 0.48, reflecting a substantial though decreasing sediment contribution from the Kalamaili Mountains. This signal aligns with the higher quartz and lower feldspar–lithic content observed in these samples, suggesting sediment maturity and longer transport pathways. The compositional data thus imply that Zh2 sandstones were dominantly derived from reworked or recycled materials sourced from the Tianshan or its foreland system. This spatial provenance contrast highlights a clear differentiation in sediment routing and source terrains during the Middle Jurassic. The Dongdaohaizi Depression acted as a localized depocenter, mainly fed by proximal uplifted blocks of the Kalamaili and Zhayier Mountains, whereas the western peripheral region (Zh2) was influenced by more distal sediment fluxes from the Tianshan Mountains. This pattern is consistent with the regional paleogeographic reconstructions, which indicate active tectonism and uplift in the eastern and southern margins of the Junggar Basin during the Middle Jurassic. Furthermore, the relatively higher feldspar contents in the C1 and CX2 samples (~26–29%) compared to Zh2 (~20%) suggest shorter sediment transport distances and more rapid deposition within the depression. The lithic content, though generally low across the dataset, is slightly elevated in CX2, hinting at episodic higher-energy input from the Zhayier structural belt. Altogether, the data support a multi-source model, with source dominance transitioning from Kalamaili in the east to Zhayier in the west, reflecting both tectonic configuration and sediment dispersal patterns across the Dongdaohaizi Depression during the Middle Jurassic.

The ternary mixing model of heavy minerals reveals a clear spatial differentiation in sediment sources across the Dongdaohaizi Depression during the Middle Jurassic (Figure 7b; Table A8 and Table A9). Samples from CX2AVE, derived from the central part of the depression, exhibit a strikingly high contribution from the Kalamaili Mountains (λ = 0.9926), with negligible input from the other two source areas. This indicates a dominant influence from the northern uplift, consistent with northward paleocurrent directions and proximity to the Kalamaili fold-and-thrust belt. Sample C1AVE, while also from within the depression but on the western side, shows a more complex provenance pattern. It incorporates substantial input from the Zhayier Mountains (λ = 0.5926) and the Kalamaili Mountains (λ = 0.3159), with minor contribution from the Tianshan source (λ = 0.0915). This suggests a sediment mixing zone influenced by both northern and central uplifts, likely facilitated by regional fluvial systems or local reworking of earlier deposits. This implies that the Zhayier orogenic belt, situated to the west, served as the second primary sediment source in the western peripheral regions of the depression, possibly through southwest-directed paleo-drainage or fan delta systems. These results collectively demonstrate a spatially variable sediment routing system within the Dongdaohaizi Depression, wherein the Kalamaili Mountains exerted a prevailing influence on the central depression fill, while the Zhayier mountains contributed more locally or peripherally to the depression.

The quantitative provenance analysis of REE reveals that the Middle Jurassic Sangonghe Formation sandstones in the Dongdaohaizi Depression are predominantly derived from the Kalamaili Mountain region, with significant contributions from the Zhayier Mountains and a lesser input from the Tianshan Mountains (Figure 7c; Table A10 and Table A11). This result aligns with the regional paleogeographic framework and sediment transport pathways. The dominant contribution from the Kalamaili Mountains (46.6–71.8%) suggests that this uplift, located to the northeast of the Dongdaohaizi Depression, was a major sediment source during the Middle Jurassic. The Kalamaili orogenic belt is composed primarily of Paleozoic arc-related igneous and metamorphic rocks, which are enriched in LREEs, especially La and Ce—consistent with the high LREE content observed in the CX2AVE and C1AVE samples. The Zhayier Mountains (24.8–40.0% contribution) represent a structurally complex zone composed of ophiolitic and island arc fragments, the moderate LREE signature of which suggests limited but geochemically distinctive sediment input. This is consistent with the intermediate Pr and Nd concentrations observed in the samples. The Tianshan Mountains contribute the least, which is geologically reasonable given their greater distance and potential sediment routing barriers during the Jurassic. The Tianshan orogen is dominated by older crystalline basement and exhibits lower overall LREE concentrations, as reflected in the REE profile of the Zh2AVE sample, which shows distinctly lower La and Ce values. Together, these results indicate a multi-source sediment supply with spatially variable input intensities. The strong LREE signature across the samples, particularly the elevated La and Ce concentrations in CX2AVE, reinforces the dominance of felsic upper crustal material, likely sourced from the active eastern orogenic belts.

The PCA analyses of the Dongdaohaizi Depression’s Sangonghe Formation sediments primarily reflect a bimodal provenance. The Kalamaili Mountains are the dominant source, evidenced by high Zr-Grt-GZi in C1/CX2 (PC1 > 0) and Th/Sc (0.71–0.72) ratios matching Kalamaili’s felsic granitoids. The Zhayier Mountains are a secondary contributor, indicated by Zh2′s elevated (La/Yb)N (1.3) and ATi (0.82), which mirror Zhayier’s mixed sedimentary–volcanic lithologies. The absence of Tianshan affinity (low δEu in C1/CX2) argues against significant input from this region. Notably, Zh2′s divergent PC2 signature implies localized western sediment routing, possibly via a paleo-channel bypassing the depression. These findings align with paleocurrent data (NW–SE flow) and regional Jurassic uplift patterns, confirming Kalamaili as the principal sediment supplier during Sangonghe deposition.

Based on the above, the statistical results from weight analysis and principal component analysis reinforce the argument that the primary provenance of the Dongdaohaizi Depression in Sangonghe Formation is derived from the Kalamaili Mountains, with additional influences from other directions (predominantly the Zhayier Mountains).

### 4.3. Tectono-Sedimentary Evolution of the Dongdaohaizi Region

REE and trace element results suggest that the sediment source regions for the Middle Jurassic Dongdaohaizi Depression reflect tectonic environments transitional between passive continental margins and continental island arcs. Previous research indicates that the onset of subduction of the Paleo-Asian Ocean (i.e., Kalamaili Ocean) transformed the eastern Junggar region from a passive margin into an arc-related setting [91,92,93]. The Kalamaili arc characteristics are geochronologically constrained to the Middle Devonian to Early Carboniferous (ca. 420–360 Ma), followed by prolonged compressional orogenesis during the Jurassic that shaped the modern basin–mountain framework [94,95,96].

During this time, the Dongdaohaizi Depression and adjacent areas underwent changes in structural and sedimentary environments. A widespread anoxic lacustrine system characterized the depositional setting in the Early to Middle Jurassic [97,98,99]. Influenced by the Indosinian and Yanshanian orogenies, a previously gridded basin structure was infilled, and widespread marshes developed as lake basins contracted. The tectonically controlled marginal swamp–lake environments efficiently facilitated sediment delivery from peripheral orogens.

**Figure 8:**
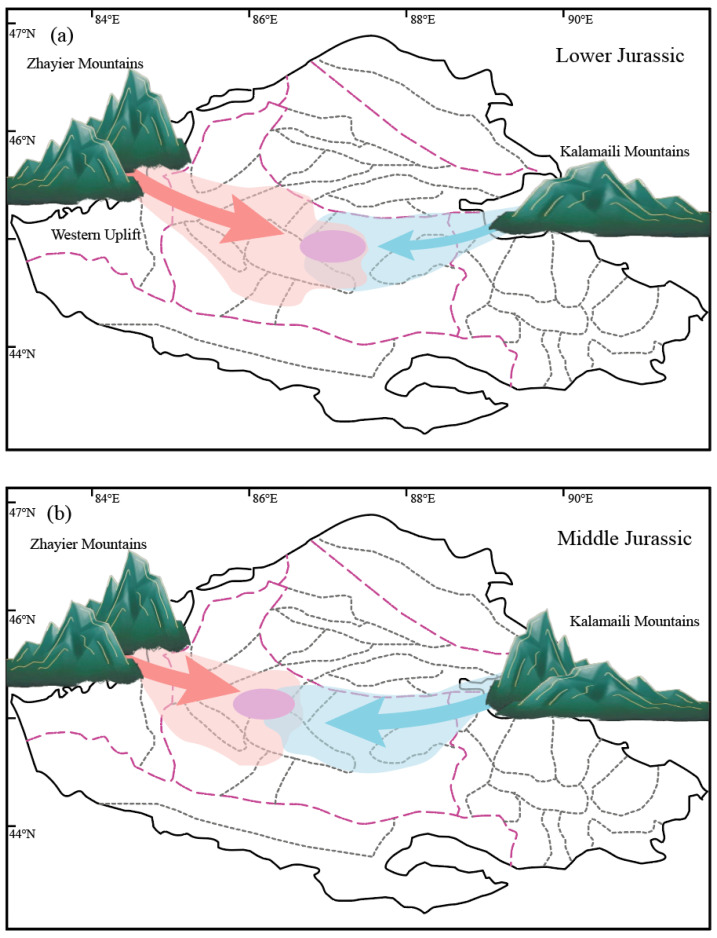
Simplified schematic model of the sedimentary and structural evolution of the Dongdaohaizi Depression and adjacent areas in the (**a**) Early Jurassic and (**b**) Middle Jurassic.

In the Early Jurassic, the Zhayier Mountains in the west and the Kalamaili Mountains in the east served as distinct sediment sources. The western source was stronger, with fluvial and tidal processes transporting sediments into the depression. Consequently, the Dongdaohaizi area and its surroundings accumulated thick sediment packages, with the main subsidence center located west of the depression. The eastern depression experienced comparatively weaker subsidence (Figure 8a). The Mosowan Uplift may have partially obstructed material exchange between the two source systems, where it demarcates distinct eastern and western provenance domains [100,101,102].

By the Middle Jurassic, renewed basement subsidence and intensified lake transgressions led to increased peat swamp deposition. Continued thrusting of peripheral fold belts into the basin caused a shift in lake center positions (Figure 8b). A predominantly arid fluvial–deltaic depositional environment emerged, favoring rapid sediment transport and accumulation [62,68,103]. The provenance distinction between eastern and western sources remained clear, though the Kalamaili source became increasingly dominant. Sedimentary thickness within the Dongdaohaizi Depression surpassed that of the western basin, and the overlapping zone between the two source areas broadened. The subsidence center gradually migrated into the western part of the depression, while the former western subsidence zone outside the depression diminished. Prolonged erosion of the Mosowan Uplift reduced its role as a barrier between the two provenance domains, facilitating greater sediment mixing.

Following the Middle Jurassic, the Junggar Basin entered a phase of intensified intracontinental deformation driven by the continued closure of the Paleo-Asian Ocean and subsequent far-field effects of the ongoing accretion and convergence between the Siberian, Kazakhstan, and Tarim blocks. During the Late Jurassic to Early Cretaceous, the basin underwent substantial tectonic reorganization characterized by enhanced compressional stress, basement reactivation, and renewed uplift of peripheral orogenic belts [62,104,105]. These processes promoted flexural subsidence in the basin interior, giving rise to widespread foreland basin-style sedimentation. In the Dongdaohaizi Depression, this tectonic transition was manifested as increased subsidence rates, accompanied by a shift from lacustrine-dominated depositional systems to more extensive fluvial and alluvial fan environments [62,68,103]. The sedimentary record reveals a coarsening–upward trend, reflecting proximity to rejuvenated source regions along both the eastern Kalamaili and western Zhayier Mountains. The progressive unroofing of these orogenic belts supplied abundant clastic material, leading to thick successions of conglomerates and sandstones that filled structural lows.

By the Early Cretaceous, the structural configuration of the Dongdaohaizi Depression began to resemble its modern geometry, with sedimentary depocenters migrating toward structurally controlled subsidence zones. The transition from syn-orogenic to post-orogenic sedimentation marked the waning influence of Paleozoic collisional dynamics and the emergence of a stable cratonic interior punctuated by episodic tectonic reactivation [63,106,107]. These findings underscore the complex interplay between tectonic inheritance, source area uplift, and climatic forcing in shaping the long-term depositional evolution of the eastern Junggar Basin.

## 5. Materials and Methods

### 5.1. Detrital Composition Analysis

The provenance characteristics of clastic sediments were investigated through systematic petrographic analysis of sandstone samples [47,108,109]. Following standard sedimentological procedures, representative samples were selected from fresh outcrops and drill cores, with all weathered surfaces carefully removed prior to thin section preparation. Thin sections (30 μm thickness) were analyzed using the Gazzi–Dickinson point-counting method, which provides reliable compositional data by minimizing grain-size effects [110]. A minimum of 500 points were counted per thin section to ensure statistical significance, focusing on detrital grains within the 0.0625–2 mm size range while excluding matrix material. For larger lithic fragments (>2 mm), individual mineral components were counted separately to maintain compositional accuracy. The detrital components were identified into quartz, feldspar and lithic fragments. This detailed classification scheme enables robust discrimination between different source rock types and tectonic provenances when matched with standard QFL ternary classifications [4,111,112]. Representative samples were selected from fresh core intervals of wells C1, CX1, and Zh2, covering all three stratigraphic members and ensuring both vertical and lateral coverage (Figure 2b).

### 5.2. Heavy Mineral Analysis

Heavy mineral assemblages were analyzed to provide additional constraints on sediment provenance, taking advantage of their diagnostic stability during transport and burial [113,114,115,116]. Sample preparation began with careful crushing of rock samples followed by sieving to isolate the 63–250 μm fraction, which optimizes heavy mineral recovery while minimizing hydraulic sorting effects. Density separation was performed using sodium polytungstate heavy liquid (ρ = 2.85 g/cm^3^), with subsequent magnetic separation using a Frantz isodynamic separator to concentrate specific mineral groups. Heavy mineral identification was conducted through combined transmitted and reflected light microscopy, supplemented by SEM-EDS analysis for ambiguous grains. Emphasis was placed on ultra-stable heavy minerals (zircon, tourmaline, rutile) and index minerals (garnet, epidote, chrome spinel) diagnostic of metamorphic and igneous sources [117,118,119]. A minimum of 200 transparent grains was counted per sample. Diagnostic indices such as the Garnet–Zircon Index (GZi) and Apatite–Tourmaline Index (ATi) were calculated to assess relative source contributions. Reproducibility was checked through duplicate splits of ~10% of the samples, with counting uncertainties maintained below ±5%.

### 5.3. Whole-Rock Geochemical Analysis

Whole-rock geochemistry was employed to establish provenance fingerprints based on immobile element distributions [120,121]. Trace element analysis of whole rock was carried out at Nanjing FocuMS Technology Co., Ltd. About 40 mg powder was mixed with 0.5 mL 60 wt% HNO_3_ and 1.0 mL 40% HF in high-pressure PTFE bombs. These bombs were steel-jacketed and placed in the oven at 195 °C for 3 days to ensure complete digestion. After cooling, the bombs were opened, dried down on a hotplate, re-dissolved with 5 mL 15 wt% HNO_3_ and 1 mL Rh internal standard, then sealed and placed in the oven at 150 °C overnight. Aliquots of the digestions (dilution factor 2000) were nebulized into an Agilent Technologies 7700x quadrupole ICP-MS (Tokyo, Japan) to determine trace elements. Geochemical reference materials of USGS: basalt (BCR-2, BHVO-2), andesite (AVG-2), rhyolite (RGM-2), granodiorite (GSP-2) were treated as quality control. Measured values of these reference materials were compared with preferred values in GeoReM database (Jochum and Nohl, 2008; http://georem.mpch-mainz.gwdg.de, accessed on 13 May 2025). Deviations were better than 20% for trace elements between 0.5~5 ppm, better than 10% for those between 5~50 ppm, and better than 5% for these exceeding 50 ppm. Trace element and REE concentrations were determined using high-resolution ICP-MS (Thermo iCAP Q, Thermo Fisher Scientific, Waltham, MA, USA), with analytical accuracy monitored through repeated measurements of international rock standards (AGV-2, GSP-2) and method blanks. The resulting data were processed using chondrite-normalized REE patterns, elemental ratio calculations (La/Sc, Th/Co, Eu/Eu*), and tectonic discrimination diagrams to identify source rock characteristics and tectonic settings. Principal component analysis was applied to the complete geochemical dataset to reveal underlying provenance relationships that might not be apparent from individual element concentrations [54,122,123]. This multi-proxy approach provides complementary lines of evidence for comprehensive provenance reconstruction while accounting for potential post-depositional modifications to the sedimentary record [124]. REE data in this study were normalized to the North American Shale Composite (NASC) [125,126,127]. Data quality was assured by analyzing international rock standards (AGV-2, GSP-2, BCR-2), internal laboratory controls, and procedural blanks. Analytical uncertainties were typically <5% (RSD) for trace elements and <3% for REEs. Detection limits ranged from 0.01 to 0.1 ppm, depending on the element. External reproducibility was monitored through replicate sample runs, yielding R^2^ > 0.99 for key elements.

To ensure the reliability and accuracy of the rare earth element (REE) data, rigorous quality assurance and quality control (QA/QC) procedures were employed throughout the ICP-MS analysis. Replicate measurements were performed on approximately 10% of the total sample set to assess instrument reproducibility. Procedural blanks were included in each batch to detect and correct for potential contamination. The relative standard deviations (RSDs) for REE concentrations in replicate analyses were typically below 5%, indicating high analytical precision. Recovery rates for the reference materials generally ranged from 90% to 110%, demonstrating good analytical accuracy.

### 5.4. Multivariate Statistical Analysis

To integrate heavy-mineral assemblages with bulk geochemical data and evaluate their joint provenance signatures, we performed principal component analysis (PCA) using R software (v4.3.1) with the FactoMineR and factoextra packages. The input dataset included 15 variables (Zr, Grt, Rt, Ap, Tur, GZi, ATi, Q, F, L, ∑REE, (La/Yb)N, δEu, Th/Sc, Zr/Sc) across n = 3 samples (C1, CX2, Zh2). Prior to PCA, the data were standardized (Z-score normalization) to ensure equal weighting of variables with disparate units.

PCA was conducted on the correlation matrix to account for variable scaling. The significance of principal components (PCs) was assessed via scree plot analysis and Kaiser’s criterion (eigenvalues > 1). We retained the top three PCs, which cumulatively explained 93.8% of the total variance (PC1: 49.6%, PC2: 35.2%, PC3: 8.96%). Loadings were calculated to quantify the contribution of each variable to the PCs, with absolute values > 0.7 considered highly influential. Results were calculated to interpret geochemical–mineralogical relationships.

Key elemental ratios such as La/Sc, Th/Co, and Eu/Eu* were calculated to identify source compositions and assess sediment recycling. Additionally, geochemical tectonic discrimination diagrams were used to infer provenance settings. Multivariate approach allows the integration of petrographic, mineralogical, and geochemical signals to construct a robust provenance model.

## 6. Conclusions

(1) The Middle Jurassic Sangonghe Formation in the Dongdaohaizi Depression is predominantly composed of feldspathic lithic sandstones and lithic sandstones, with minor lithic–feldspathic components. Heavy mineral assemblages dominated by zircon, garnet, chromite, and rutile suggest provenance from a complex lithologic background, including intermediate to felsic igneous, metamorphic, and mafic source rocks.

(2) Geochemical data show that the total REE contents of the sandstones range from 101.84 to 192.68 μg/g, with ∑LREE/∑HREE ratios between 6.59 and 13.25. The average δEu and δCe values are near-neutral to weakly negative, respectively, indicating weak or no europium and cerium anomalies. These patterns suggest stable source signatures and limited post-depositional alteration.

(3) Provenance discrimination diagrams based on trace elements (La-Th-Sc, Th-Co-Zr/10, Th-Sc-Zr/10, La/Y-Sc/Cr) indicate that the majority of samples were derived from continental island arc settings, with minor contributions from passive continental margin sources.

(4) A dual-provenance system is identified: the eastern Kalamaili Mountains and the western Zhayier Mountains. In the Early Jurassic, the Zhayier source was dominant, delivering sediments through fluvial and tidal systems to the western part of the depression. However, by the Middle Jurassic, enhanced thrusting along surrounding fold belts and sustained erosion of the Mosowan Uplift facilitated a shift in the depocenter westward to the exterior of the Dongdaohaizi Depression and increased mixing of eastern and western source contributions.

(5) These findings highlight the dynamic interplay between tectonic activity, sediment routing systems, and structural barriers in controlling provenance and sediment dispersal within the eastern Junggar Basin during the Jurassic. The results provide important constraints for reconstructing paleogeographic evolution and sedimentary basin dynamics in the southern Central Asian Orogenic Belt.

## Figures and Tables

**Table 1:** Average geochemical parameters of rare earth elements in samples from the Sangonghe Formation.

Sample and Strata	ΣREE	LREE	HREE	LREE/HREE	La_N_/Yb_N_	δEu	δCe	La_N_/Sm_N_	Gd_N_/Yb_N_	Ceanom
C1-J_2_s_3_	191.30	168.45	22.85	7.37	0.98	1.08	0.96	1.01	0.96	−0.03
C1-J_2_s_2_	182.73	159.56	23.18	6.88	0.96	1.12	0.96	1.07	0.94	−0.01
C1-J_2_s_1_	187.88	166.90	20.99	7.95	0.98	1.01	0.96	1.08	0.79	−0.04
Cx2-J_2_s_2_	190.89	177.30	13.59	13.05	2.01	1.10	0.90	1.77	0.85	−0.07
Zh2-J_2_s_2_	224.19	203.51	20.68	9.84	1.30	1.10	1.01	0.87	1.15	0.02

## Data Availability

All data supporting the reported results can be found at https://www.mdpi.com.

## Appendix A

**Table A1:** Statistic of clastic materials in samples from the Sangonghe Formation.

Strata	Sample	Quartz (%)	Feldspar (%)	Lithic Fragments (%)	Lava Rocks (%)	Metamorphic Rocks (%)	Sedimentary Rocks (%)	Maturity
J_1_s_2_	C1-01	39.3	21.7	39.0	41.5	50.0	7.4	0.65
	C1-02	43.3	19.6	37.2	41.5	45.0	13.5	0.76
	C1-03	43.3	17.6	39.1	41.5	45.0	13.5	0.76
	C1-04	47.6	17.6	34.8	45.7	49.5	4.9	0.91
	C1-05	47.6	19.4	33.1	45.7	44.5	9.8	0.91
	C1-06	52.3	21.3	26.4	41.1	49.0	9.9	1.10
	C1-07	47.1	19.2	33.7	41.1	44.1	14.8	0.89
	C1-08	47.1	17.3	35.6	45.2	44.1	10.7	0.89
	C1-09	51.8	15.5	32.7	40.7	44.1	15.2	1.08
	C1-10	51.8	17.1	31.1	36.6	48.5	14.9	1.08
	CX2-01	45.3	13.5	41.3	71.4	19.1	9.5	0.83
	CX2-02	49.8	13.5	36.7	78.6	17.1	4.3	0.99
	CX2-03	44.8	13.3	41.8	70.7	21.0	8.3	0.81
	CX2-04	44.8	13.3	41.8	63.6	21.0	15.4	0.81
	CX2-05	49.3	14.6	36.0	70.0	19.1	10.9	0.97
	CX2-06	44.4	16.1	39.5	77.0	19.1	3.9	0.80
	CX2-07	49.8	12.1	38.1	78.6	19.1	2.4	0.99
	CX2-08	49.3	14.8	35.9	70.0	17.1	12.8	0.97
	CX2-09	44.4	16.3	39.3	77.0	17.1	5.8	0.80
	CX2-10	39.9	13.2	46.9	52.1	21.0	27.0	0.67
	Zh2-01	55.0	11.4	33.7	73.5	17.7	8.8	1.22
	Zh2-02	49.5	12.5	38.0	80.9	17.7	1.5	0.98
	Zh2-03	60.4	12.5	27.0	73.5	19.4	7.1	1.53
	Zh2-04	55.0	10.3	34.8	73.5	19.4	7.1	1.22
	Zh2-05	55.0	10.3	34.8	80.9	19.4	0.3	1.22
	Zh2-06	60.4	12.5	27.0	80.9	15.9	3.2	1.53
	Zh2-07	60.4	11.4	28.2	66.2	19.4	14.4	1.53
	Zh2-08	60.4	12.5	27.0	80.9	19.4	0.3	1.53
	Zh2-09	60.4	10.3	29.3	66.2	19.4	14.4	1.53
	Zh2-10	60.4	11.4	28.2	66.2	15.9	17.9	1.53
	Zh2-11	34.3	18.4	47.3	64.4	22.0	13.6	0.52
	Zh2-12	37.7	20.3	42.0	58.0	24.2	17.8	0.61
	Zh2-13	30.8	18.4	50.7	58.0	22.0	20.0	0.45
	Zh2-14	34.3	20.3	45.4	70.9	19.8	9.4	0.52
	Zh2-15	37.7	16.6	45.7	58.0	19.8	22.2	0.61
	Zh2-16	37.7	20.3	42.0	58.0	24.2	17.8	0.61
	Zh2-17	30.8	20.3	48.9	70.9	19.8	9.4	0.45
	Zh2-18	34.3	20.3	45.4	70.9	24.2	5.0	0.52
	Zh2-19	34.3	20.3	45.4	70.9	22.0	7.2	0.52
	Zh2-20	34.3	16.6	49.1	58.0	22.0	20.0	0.52

**Table A2:** Relative content of heavy minerals in samples from the Sangonghe Formation.

Strata	Sample	Heavy Mineral (%)									GZi	ATi
		Zircon	Garnet	Picotite	Tourmaline	Apatite	Rutile	Anatase	Leucoxene	Other		
J_1_s_2_	C1-01	30.29	28.65	19.78	4.17	0.34	0.87	5.95	1.44	8.51	48.61	7.54
	C1-02	30.74	30.42	19.81	4.22	0.36	0.96	5.73	1.47	6.30	49.74	7.86
	C1-03	29.84	31.01	20.26	4.15	0.36	0.94	5.59	1.44	6.40	50.96	7.98
	C1-04	30.38	29.36	19.85	4.24	0.35	0.90	6.08	1.52	7.33	49.15	7.63
	C1-05	29.39	33.77	19.18	4.34	0.33	0.88	5.46	1.58	5.07	53.47	7.07
	C1-06	29.96	30.81	21.04	4.04	0.35	0.93	5.92	1.51	5.44	50.70	7.97
	C1-07	32.24	31.25	19.00	4.11	0.36	0.94	5.85	1.46	4.79	49.22	8.05
	C1-08	32.78	31.18	18.92	4.33	0.36	0.88	5.99	1.48	4.07	48.75	7.68
	C1-09	29.29	30.24	20.53	3.99	0.34	0.93	5.51	1.53	7.65	50.80	7.85
	C1-10	32.11	33.35	19.82	4.15	0.33	0.87	5.85	1.38	2.14	50.95	7.37
	AVE	30.34	31.39	19.88	4.19	0.35	0.9	5.83	1.49	5.63	50.85	7.71
	CX2-01	34.85	28.88	9.30	0.25	0.54	0.80	0.85	0.17	24.36	45.32	68.35
	CX2-02	32.81	28.92	10.36	0.28	0.52	0.82	0.85	0.17	25.27	46.85	65.00
	CX2-03	32.30	28.61	9.67	0.26	0.57	0.83	0.76	0.18	26.82	46.97	68.67
	CX2-04	32.11	28.89	9.30	0.27	0.56	0.88	0.80	0.18	27.00	47.36	67.47
	CX2-05	32.32	27.01	10.35	0.27	0.57	0.80	0.77	0.18	27.74	45.53	67.86
	CX2-06	31.99	29.33	10.23	0.27	0.55	0.77	0.87	0.19	25.81	47.83	67.07
	CX2-07	32.40	28.72	9.56	0.27	0.53	0.83	0.77	0.18	26.74	46.99	66.25
	CX2-08	35.02	28.16	9.83	0.27	0.54	0.84	0.86	0.18	24.31	44.57	66.67
	CX2-09	33.80	28.73	10.07	0.28	0.53	0.82	0.79	0.18	24.80	45.95	65.43
	CX2-10	32.21	28.76	10.46	0.27	0.52	0.82	0.86	0.18	25.94	47.17	65.82
	AVE	32.97	28.3	9.89	0.27	0.55	0.82	0.82	0.18	26.2	46.19	67.07
	Zh2-01	16.38	25.00	1.81	1.01	0.70	0.75	2.23	0.71	51.40	60.42	40.94
	Zh2-02	17.14	25.41	1.80	1.01	0.70	0.73	2.11	0.67	50.43	59.72	40.94
	Zh2-03	16.68	24.18	1.91	1.10	0.70	0.76	2.25	0.75	51.68	59.18	38.89
	Zh2-04	17.49	25.24	1.93	1.13	0.71	0.73	2.13	0.71	49.95	59.07	38.59
	Zh2-05	17.44	25.36	1.85	1.18	0.67	0.70	2.18	0.68	49.93	59.25	36.22
	Zh2-06	16.62	24.01	1.85	1.10	0.71	0.72	2.13	0.66	52.19	59.09	39.23
	Zh2-07	16.07	24.53	1.79	1.05	0.65	0.73	2.20	0.72	52.27	60.42	38.24
	Zh2-08	17.09	25.56	1.70	1.04	0.71	0.77	2.28	0.77	50.10	59.93	40.57
	Zh2-09	17.65	23.90	1.94	1.05	0.66	0.73	2.23	0.74	51.10	57.52	38.60
	Zh2-10	15.95	24.56	1.81	1.07	0.65	0.73	2.10	0.71	52.42	60.63	37.79
	AVE	16.66	25.37	1.83	1.08	0.68	0.72	2.18	0.72	50.76	60.36	38.64

**Table A3:** Analysis data of rare earth elements in samples from the Sangonghe Formation.

Strata	Sample	Element (μg/g)													
		La	Ce	Pr	Nd	Sm	Eu	Gd	Tb	Dy	Ho	Er	Tm	Yb	Lu
Cx2-J1s2-3	CX2-01	49.86	81.24	9.19	34.27	5.48	1.03	3.53	0.61	3.57	0.69	2.29	0.35	2.34	4.77
	CX2-02	49.61	81.88	9.25	35.10	5.27	1.00	3.23	0.58	3.71	0.72	2.29	0.33	2.28	4.88
	CX2-03	49.18	76.41	8.99	32.79	5.18	0.98	3.14	0.55	3.47	0.71	2.31	0.33	2.24	4.64
	CX2-04	48.02	80.77	9.30	32.72	5.44	1.01	3.31	0.56	3.72	0.73	2.10	0.32	2.35	4.93
	CX2-05	47.74	80.32	9.66	33.37	5.44	0.99	3.22	0.56	3.59	0.79	2.23	0.35	2.36	4.76
	CX2-06	47.04	84.26	9.39	34.43	5.28	0.97	3.26	0.59	3.70	0.72	2.30	0.34	2.34	4.97
	CX2-07	50.84	80.58	9.11	33.42	5.71	1.02	3.35	0.58	3.84	0.69	2.31	0.37	2.26	5.03
	CX2-08	50.70	85.17	9.12	33.92	5.33	1.01	3.25	0.59	3.54	0.71	2.31	0.34	2.35	5.02
	CX2-09	48.17	80.28	9.20	31.09	4.98	0.98	3.36	0.57	3.63	0.71	2.24	0.34	2.26	4.77
	CX2-10	47.25	82.92	9.04	34.23	4.98	0.99	3.18	0.56	3.62	0.73	2.18	0.33	2.35	4.80
	AVE	49.20	81.10	9.25	33.30	5.22	0.98	3.33	0.56	3.63	0.73	2.28	0.34	2.30	4.92
Cx2-J1s2-2	CX2-11	49.16	80.38	9.82	31.85	5.28	1.02	3.33	0.58	3.80	0.68	2.23	0.36	2.28	4.90
	CX2-12	49.57	81.00	9.35	31.98	4.87	1.01	3.66	0.59	3.87	0.73	2.19	0.34	2.18	4.85
	CX2-13	45.78	80.43	9.35	29.83	4.92	1.01	3.33	0.57	3.75	0.72	2.30	0.35	2.30	4.53
	CX2-14	50.48	81.70	9.09	32.93	4.97	1.01	3.36	0.59	3.92	0.69	2.17	0.34	2.36	4.88
	CX2-15	48.50	81.21	9.48	32.44	5.21	1.02	3.49	0.54	3.88	0.72	2.28	0.35	2.40	4.90
	CX2-16	50.40	78.77	9.55	32.14	5.12	1.04	3.38	0.58	3.59	0.68	2.25	0.32	2.36	5.05
	CX2-17	48.69	78.14	9.00	33.59	5.08	1.02	3.35	0.55	3.66	0.71	2.11	0.34	2.26	4.92
	CX2-18	48.03	80.83	9.10	32.09	5.20	0.98	3.31	0.61	3.76	0.72	2.08	0.34	2.21	5.08
	CX2-19	47.51	78.04	8.89	31.39	5.00	0.95	3.28	0.56	3.72	0.69	2.30	0.32	2.36	4.80
	CX2-20	45.79	80.45	8.67	31.76	5.02	0.99	3.09	0.56	3.75	0.73	2.27	0.32	2.36	5.00
	AVE	48.80	80.80	9.16	32.40	5.17	0.99	3.41	0.57	3.70	0.73	2.20	0.34	2.29	4.88
C1-J1s3	C1-11	36.84	79.61	8.85	33.73	7.16	1.50	6.07	0.98	6.30	1.20	3.53	0.55	3.78	3.87
	C1-12	39.67	76.50	9.10	33.92	7.04	1.60	6.52	0.98	6.09	1.16	3.42	0.54	3.73	4.01
	C1-13	37.00	76.88	9.86	34.30	7.22	1.57	6.38	0.96	6.04	1.20	3.47	0.53	3.72	3.98
	C1-14	37.62	80.14	8.90	33.32	7.06	1.65	6.19	0.90	5.91	1.24	3.45	0.53	3.79	4.19
	C1-15	36.78	79.63	9.24	34.51	6.86	1.62	6.40	0.96	6.01	1.16	3.58	0.53	3.50	3.76
	C1-16	36.84	77.75	9.19	35.51	7.07	1.54	6.31	0.95	6.09	1.24	3.47	0.50	3.76	3.92
	C1-17	39.86	76.69	9.74	36.89	7.07	1.59	5.85	0.96	6.10	1.21	3.72	0.55	3.68	3.91
	C1-18	41.54	75.99	9.48	34.38	6.95	1.50	6.10	0.91	5.96	1.22	3.49	0.59	3.95	3.80
	C1-19	39.84	77.67	9.05	36.66	7.02	1.59	6.78	0.98	5.61	1.22	3.62	0.54	3.86	3.72
	C1-20	38.38	76.42	8.95	34.63	7.39	1.57	6.12	0.95	5.90	1.25	3.50	0.50	3.70	3.99
	AVE	38.60	77.00	9.29	34.80	7.15	1.53	6.24	0.96	6.07	1.20	3.55	0.54	3.71	3.86
C1-J1s2-3	C1-01	34.51	68.05	7.63	30.34	6.87	1.48	5.98	0.87	6.14	1.16	3.59	0.52	3.63	3.52
	C1-02	32.69	63.69	7.79	30.80	6.81	1.53	5.70	0.92	6.00	1.24	3.74	0.59	3.81	3.30
	C1-03	34.04	69.37	8.11	32.07	6.21	1.57	5.91	0.90	5.92	1.14	3.69	0.56	3.72	3.46
	C1-04	33.29	62.86	7.79	29.09	6.42	1.47	6.04	0.92	6.67	1.16	3.80	0.55	3.68	3.42
	C1-05	33.64	62.64	8.03	31.48	6.20	1.55	6.13	0.92	6.19	1.20	3.73	0.58	3.90	3.45
	C1-06	33.70	66.69	8.05	30.28	6.31	1.45	6.26	0.95	6.48	1.23	3.83	0.58	3.87	3.33
	C1-07	34.26	68.53	8.20	29.76	6.22	1.38	6.06	0.91	6.30	1.20	3.75	0.54	3.91	3.56
	C1-08	32.68	66.94	8.40	31.28	6.67	1.56	5.98	0.90	6.28	1.23	3.69	0.55	3.88	3.38
	C1-09	35.47	67.66	7.46	31.29	6.30	1.41	5.88	0.96	6.23	1.24	3.94	0.56	3.98	3.34
	C1-10	34.04	65.77	8.23	31.30	6.97	1.49	6.45	0.93	5.84	1.15	3.82	0.54	3.78	3.31
	AVE	33.60	66.90	8.05	30.70	6.46	1.51	6.05	0.94	6.14	1.21	3.70	0.55	3.86	3.36
C1-J1s1	C1-21	40.28	73.08	8.73	35.10	7.17	1.32	5.25	0.78	5.33	1.10	3.69	0.51	3.92	3.95
	C1-22	38.62	76.45	9.31	34.24	6.86	1.24	5.23	0.85	5.42	1.12	3.70	0.51	3.89	3.84
	C1-23	38.95	78.99	9.29	35.78	6.53	1.33	5.10	0.81	5.30	1.15	3.59	0.53	3.68	3.88
	C1-24	39.53	77.17	8.62	33.36	6.60	1.24	5.39	0.83	5.04	1.11	3.78	0.52	3.67	3.85
	C1-25	39.09	80.57	8.95	35.51	6.90	1.18	5.01	0.84	5.47	1.07	3.53	0.54	3.60	3.79
	C1-26	41.79	76.58	8.87	36.49	6.79	1.29	5.06	0.84	5.78	1.15	3.62	0.53	3.74	3.83
	C1-27	39.09	74.61	9.40	32.75	6.69	1.30	5.12	0.78	5.48	1.21	3.59	0.52	3.96	3.86
	C1-28	38.56	72.58	9.18	34.60	6.83	1.30	5.23	0.82	5.71	1.13	3.72	0.53	3.66	3.72
	C1-29	39.26	71.20	9.07	33.32	6.69	1.36	4.91	0.82	5.35	1.18	3.58	0.53	3.51	3.70
	C1-30	36.40	74.94	9.45	36.20	6.41	1.30	4.89	0.84	5.04	1.14	3.46	0.53	3.58	3.84
	AVE	38.70	76.30	9.18	34.70	6.72	1.27	5.14	0.81	5.51	1.14	3.57	0.53	3.72	3.87
Zh2-J1s	Zh2-01	19.31	36.54	4.32	17.05	3.48	0.80	3.65	0.49	3.22	0.69	2.10	0.30	1.92	0.32
	Zh2-02	18.51	37.53	4.18	15.94	3.53	0.79	3.46	0.51	3.37	0.72	2.04	0.28	2.01	0.33
	Zh2-03	19.30	36.20	4.45	16.22	3.41	0.82	3.63	0.51	3.45	0.68	2.02	0.28	2.16	0.34
	Zh2-04	18.67	33.79	4.30	16.69	3.43	0.80	3.47	0.52	3.35	0.68	1.92	0.29	2.01	0.32
	Zh2-05	19.19	37.59	4.12	16.75	3.57	0.75	3.41	0.53	3.10	0.73	2.05	0.28	1.99	0.32
	Zh2-06	20.10	35.10	4.34	16.88	3.25	0.75	3.35	0.50	3.34	0.70	1.96	0.31	1.99	0.34
	Zh2-07	20.18	34.57	4.46	16.07	3.33	0.79	3.56	0.53	3.34	0.69	2.11	0.29	2.02	0.31
	Zh2-08	19.20	36.76	4.37	16.13	3.47	0.75	3.52	0.53	3.29	0.72	2.01	0.27	1.93	0.31
	Zh2-09	18.73	38.33	4.36	15.51	3.22	0.79	3.52	0.53	3.20	0.70	2.04	0.31	2.08	0.30
	Zh2-10	19.42	35.29	4.39	17.11	3.42	0.79	3.65	0.55	3.25	0.71	2.00	0.28	1.97	0.32
	AVE	19.10	36.40	4.34	16.80	3.44	0.78	3.46	0.51	3.25	0.70	2.00	0.29	2.01	0.31

**Table A4:** Major parameters selected as inputs for principal component analysis.

Sample ID	Zr	Grt	Rt	Ap	Tur	GZi	ATi	Q	F	L	∑REE	(La/Yb)_N	δEu	Th/Sc	Zr/Sc
C1AVE	30.34	31.39	0.90	0.35	4.19	50.85	7.71	47.12	18.63	34.27	182.73	0.96	1.12	0.71	19.93
CX2AVE	32.97	28.30	0.82	0.55	0.27	46.19	67.07	39.90	13.20	46.90	190.89	2.01	1.10	0.72	19.44
Zh2AVE	16.66	25.37	0.72	0.68	1.08	60.36	38.64	34.30	16.60	49.10	224.19	1.30	1.10	0.69	19.25

**Table A5:** Variance explained by principal components (PCs) in the provenance analysis of the Sangonghe Formation sandstones.

Principal Component	Eigenvalue	Variance Explained (%)	Cumulative Variance (%)
PC1	7.94	49.64	49.64
PC2	5.63	35.17	84.81
PC3	1.43	8.96	93.77

**Table A6:** Average compositional data of clastic materials from the three major potential provenance regions.

	Quartz (%)	Feldspar (%)	Lithic Fragments (%)
A (Kalamaili Mountains)	39.3	21.7	39.0
B (Zhayier Mountains)	43.3	19.6	37.2
C (Tianshan Mountains)	43.3	17.6	39.1

**Table A7:** Weight analysis of representative samples versus the three major potential provenances of clastic materials.

Sample	λA	λB	λC
C1-01	0.5512	0.3917	0.0571
C1-02	0.598	0.3484	0.0536
C1-03	0.6183	0.3234	0.0583
C1-04	0.6443	0.3066	0.0492
C1-05	0.6261	0.3284	0.0455
C1-06	0.6342	0.3324	0.0334
C1-07	0.6253	0.328	0.0467
C1-08	0.6445	0.3047	0.0509
C1-09	0.6892	0.2654	0.0454
C1-10	0.6723	0.2856	0.0421
CX2-01	0.6763	0.2594	0.0644
CX2-02	0.7014	0.2447	0.054
CX2-03	0.6759	0.2582	0.0658
CX2-04	0.6759	0.2582	0.0658
CX2-05	0.6862	0.2615	0.0523
CX2-06	0.6412	0.2992	0.0596
CX2-07	0.7181	0.2245	0.0574
CX2-08	0.6838	0.2642	0.052
CX2-09	0.639	0.3019	0.0591
CX2-10	0.6458	0.275	0.0793
Zh2-01	0.7515	0.2005	0.0481
Zh2-02	0.7117	0.2313	0.057
Zh2-03	0.7616	0.2028	0.0355
Zh2-04	0.7651	0.1844	0.0505
Zh2-05	0.7651	0.1844	0.0505
Zh2-06	0.7616	0.2028	0.0355
Zh2-07	0.7742	0.1881	0.0377
Zh2-08	0.7616	0.2028	0.0355
Zh2-09	0.7873	0.1728	0.0399
Zh2-10	0.7742	0.1881	0.0377

**Table A8:** Average compositional data of heavy minerals from the three major potential provenance regions.

	Heavy Mineral (%)								
	Zircon	Garnet	Picotite	Tourmaline	Apatite	Rutile	Anatase	Leucoxene	Other
A (Kalamaili Mountains)	30	30	20	5	1	1	6	3	4
B (Zhayier Mountains)	33	28	10	1	1	1	1	1	24
C (Tianshan Mountains)	17	25	2	1	1	1	2	1	50

**Table A9:** Weight analysis of representative samples versus the three major potential provenances in terms of heavy minerals.

Sample	λA	λB	λC
C1AVE	0.9926	0.0061	0.0013
CX2AVE	0.3159	0.5926	0.0915
Zh2AVE	0.0507	0.0386	0.9107

**Table A10:** Average compositional data of REE from the three major potential provenance regions.

	La	Ce	Pr	Nd
A (Kalamaili Mountains)	33.57	54.18	6.74	26.12
B (Zhayier Mountains)	35.42	83.66	9.15	31.89
C (Tianshan Mountains)	13.08	30.31	2.39	11.44

**Table A11:** Weight analysis of representative samples versus the three major potential provenances in terms of REE.

Sample	λA	λB	λC
CX2AVE	0.59	0.40	0.01
C1AVE	0.47	0.25	0.28
Zh2AVE	0.70	0.17	0.28
